# Sex Variations in Retinal Microcirculation Response to Lower Body Negative Pressure

**DOI:** 10.3390/biology12091224

**Published:** 2023-09-11

**Authors:** Adam Saloň, Nikola Vladic, Karin Schmid-Zalaudek, Bianca Steuber, Anna Hawliczek, Janez Urevc, Andrej Bergauer, Vid Pivec, Vishwajeet Shankhwar, Nandu Goswami

**Affiliations:** 1 Division of Physiology & Pathophysiology, Otto Loewi Research Center, Medical University of Graz, 8010 Graz, Austriakarin.schmid@medunigraz.at (K.S.-Z.);; 2 Faculty of Health and Social Sciences, Inland Norway University of Applied Sciences, 2624 Lillehammer, Norway; 3 College of Medicine, Medical University of Rijeka, 51000 Rijeka, Croatia; 4 Faculty of Mechanical Engineering, University of Ljubljana, 1000 Ljubljana, Slovenia; 5 LKH Südsteiermark, 18 8435 Wagna, Austria; 6 Clinical Department for General and Abdominal Surgery, University Clinical Centre Maribor, 2000 Maribor, Slovenia; 7 College of Medicine, Mohammed Bin Rashid University of Medicine and Health Sciences, Dubai P.O. Box 505055, United Arab Emirates; 8 Department of Integrative Health, Alma Mater Europaea Maribor, 2000 Maribor, Slovenia

**Keywords:** lower body negative pressure, sex, microcirculation, retinal vessels

## Abstract

**Simple Summary:**

This study explored how retinal vasculature changes during central hypovolemia induced by lower body negative pressure (LBNP). LBNP is known to shift blood to the lower body and is routinely employed to assess the effects of central hypovolemia and/or to simulate the effects of hemorrhage on systems physiology. In this study, retinal imaging was carried out in participants of both sexes as they underwent LBNP. Surprisingly, no significant changes were observed in retinal blood flow between time points or across the sexes. This study is the first in this field, shedding light on retinal response during a moderate LBNP of −40 mmHg, which induces fluid shifts and evokes systematic physiological responses like those that occur during upright standing. However, further research is needed with stronger LBNP levels, including those that can induce pre-fainting (presyncope) states, to fully understand how retinal microcirculation adapts during complete cardiovascular collapse (e.g., during hypovolemic shock) and/or during severe hemorrhage.

**Abstract:**

Introduction: Lower body negative pressure (LBNP) is routinely used to induce central hypovolemia. LBNP leads to a shift in blood to the lower extremities. While the effects of LBNP on physiological responses and large arteries have been widely reported, there is almost no literature regarding how these cephalad fluid shifts affect the microvasculature. The present study evaluated the changes in retinal microcirculation parameters induced by LBNP in both males and females. Methodology: Forty-four participants were recruited for the present study. The retinal measurements were performed at six time points during the LBNP protocol. To prevent the development of cardiovascular collapse (syncope) in the healthy participants, graded LBNP until a maximum of −40 mmHg was applied. A non-mydriatic, hand-held Optomed Aurora retinal camera was used to capture the retinal images. MONA Reva software (version 2.1.1) was used to analyze the central retinal arterial and venous diameter changes during the LBNP application. Repeated measures ANOVAs, including sex as the between-subjects factor and the grade of the LBNP as the within-subjects factor, were performed. Results: No significant changes in retinal microcirculation were observed between the evaluated time points or across the sexes. Conclusions: Graded LBNP application did not lead to changes in the retinal microvasculature across the sexes. The present study is the first in the given area that attempted to capture the changes in retinal microcirculation caused by central hypovolemia during LBNP. However, further research is needed with higher LBNP levels, including those that can induce pre-fainting (presyncope), to fully understand how retinal microcirculation adapts during complete cardiovascular collapse (e.g., during hypovolemic shock) and/or during severe hemorrhage.

## 1. Introduction

Lower body negative pressure (LBNP) serves as a countermeasure against the headward fluid shift in microgravity during spaceflight [1]. It is also commonly used to assess an individual’s cardiovascular stability during central hypovolemia, which occurs during prolonged standing and hemorrhage. By shifting blood from the upper to lower parts, particularly the pelvis, legs, and extravascular space, LBNP reduces the central venous pressure and venous return [2]. Reported differences in orthostatic tolerance across males and females have been documented. Goswami et al. reported that women have lower central hypovolemia tolerance [3]. Although the data regarding the lower tolerance in women is established [4,5], the reasons behind this discrepancy remain partially elusive.

While the effects of LBNP on physiological responses and large arteries have been widely reported, there is almost no literature regarding how these cephalad fluid shifts affect the microvasculature. The present study evaluated retinal microcirculation parameters, especially as previous studies have reported that changes in the small vessels of the retina precede changes in larger vessels. [6,7,8,9,10,11,12,13,14,15]. While constriction of the retinal vessels is seen in hypertension [8,9,10], retinal arteriolar constriction and venular dilatation have been shown to predict coronary heart disease and stroke [11,12,13,14,15]. Retinal imaging was used in this study, as it has emerged as a valid, rapid, and cost-effective method for early cardiovascular abnormality detection, which could potentially be used during application of LBNP or in space missions. While previous studies have linked microcirculation and sepsis [16,17,18,19,20], none examined LBNP. The present study attempted to fill this research gap regarding LBNP and microcirculation by exploring how LBNP levels (−10, −20, −30, and −40 mmHg) and sex (males and females) influence retinal microcirculation in young, healthy individuals. We hypothesized that variations in retinal microcirculation parameters across the protocol (baseline, different LBNP levels, and recovery) and between the sexes would be seen.

## 2. Methods

The present study was performed at the Division of Physiology, Medical University of Graz, Austria. Approval for the study was obtained from the Ethics committee of the Medical University of Graz, Austria (Ref: EK 25-551 ex 12/13). All the data were collected in accordance with Good Clinical Practice standards, and the study design was compliant with the Declaration of Helsinki of WMA (2013). All the participants received information about the study and provided written consent prior to participation. The signed consent forms are stored at the Division of Physiology, Medical University of Graz. 

### 2.1. Sample Size Calculation 

Using the typical retinal vascular changes from our previous studies [21,22] with an error probability (α) of 0.05 and a power (1 − ß) of 0.80, we estimated that the required number of participants was 20. Considering a possible dropout rate of 20%, we recruited forty-two adult participants (20 males, 22 females; age: 24.9 ± 5.9 years; BMI: 21.8 ± 25 kg/m^2^). Of these, complete data sets were available for twenty-seven participants (*n* = 11 males; *n* = 16 females).

### 2.2. Participants

**Figure 1:**
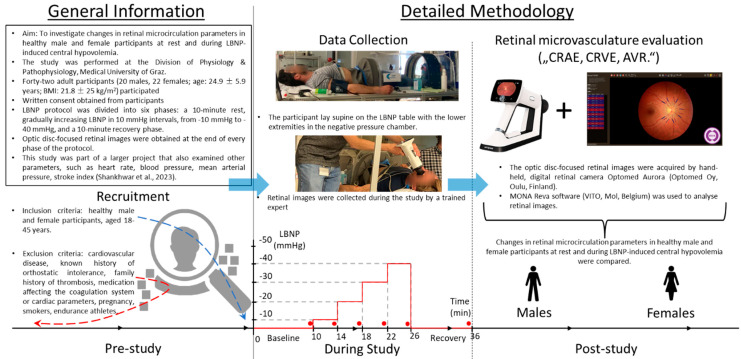
This diagram provides a concise overview of the study flow, encompassing the phase preceding the commencement of the study. A horizontal line accompanied by arrows as well as two blue arrows pointing rightward illustrates the study’s temporal progression. The “Prior study conduction” section provides foundational study details and the recruitment specifics. The “Data collection” phase within the “Study conduction” section is portrayed through two photos captured of the study and a graph depicting temporal changes in LBNP levels. The graph charts the specific LBNP levels on the *y*-axis against minutes on the *x*-axis (including baseline and recovery periods). The red line accentuates the variation of LBNP over time. The red line accentuates the variation of LBNP over time. The red dots highlight the data collection points. The final section of the diagram introduces the retinal camera and software used for capturing and assessing the retinal images. It also emphasizes the primary study objective: examining sex-based disparities in retinal parameters during central hypovolemia. The parameters considered include the central retinal arteriolar equivalent (CRAE), the central retinal venular equivalent (CRVE), and the artery-to-vein ratio (AVR).

Forty-two adult participants (20 males, 22 females; age: 24.9 ± 5.9 years; BMI: 21.8 ± 25 kg/m^2^) took part in this study. The protocol of the study was explained to the participants by the department staff. A simplified overview of the study flow can be seen in Figure 1. The detailed protocol of the study and the applied inclusion and exclusion criteria are described in Shankhwar et al. [23].

### 2.3. LBNP Protocol 

The participants were measured at six-time points: at baseline supine, four times at gradually increasing negative pressures (−10 mmHg, −20 mmHg, −30 mmHg, and −40 mmHg, LBNP was increased by 10 mmHg every 4 min), and finally, during a recovery period (Figure 1). All the measurements were performed in the same room and by the same operator at the Division of Physiology, Medical University of Graz. The parameters of the microcirculation used for evaluating cardiovascular health were collected using retinal imaging. This study was part of a larger project that also examined other parameters, such as heart rate, blood pressure, mean arterial pressure, stroke index, cardiac index, total peripheral resistance index, and low-frequency and high-frequency RRI band power derived from the heart rate variability signal [23]. 

### 2.4. Microcirculation Measurements

The optic disc-focused retinal images were acquired by a hand-held, portable 30° field-of-view digital retinal camera, Optomed Aurora (Optomed Oy, Oulu, Finland), by the same trained researcher at six time points. Another trained researcher who was blinded to the study participants analyzed the retinal images using MONA REVA software (version 2.1.1,VITO, Mol, Belgium; [24]). For further details, see Saloň et al. [21]. The retinal microcirculation parameters were as follows: central retinal arteriolar equivalent (CRAE), central retinal venular equivalent (CRVE), and artery-to-vein ratio (AVR).

### 2.5. Statistical Analysis 

All the data were tested for normality of the distribution using the Shapiro–Wilks test, and the cases that largely deviated from the group mean and/or the missing data were excluded. Due to the small number of complete data sets, only the baseline, LBNP-40, and recovery measurements were included in the statistical analyses. Repeated measures ANOVAs were performed with sex as the between-subjects factor and three evaluated time points (baseline, LBNP-40, and recovery) as within-subjects factors to analyze changes in CRAE and CRVE. For pairwise comparisons between the different conditions (baseline, LBNP40, recovery), the significance level was adjusted according to Bonferroni. All the data were analyzed using SPSS (IBM SPSS Statistics for Windows, Version 27.0., Armonk, NY, USA: IBM Corp).

## 3. Results

Six (14.3%) out of the forty-two participants dropped out (did not finish the protocol) of this study. The baseline characteristics of the participants divided based on sex are shown in Table 1. Retinal images were collected from thirty-seven participants. After the exclusion of low-quality images that were unfit for analysis, complete datasets were available for *n* = 27 participants. All the results in the present manuscript were obtained by analyzing those 27 participants (Table 1). 

### Retinal Microcirculation Measurements

Table 2 displays the values of the retinal microvascular parameters for three analyzed measurement time points divided based on sex.

Considering the CRAE, no significant changes between the baseline, LBNP-40, and recovery (F (2,50) = 0.579, *p* = 0.564) were found. Although the females showed higher values of CRAE, the effect was not significant (F (1,25) = 2.231, *p* = 0.148). The interaction between LBNP and sex was also found to not be significant (F (2,50) = 0.088, *p* = 0.916). 

Like CRAE, there were no significant changes between the baseline, LBNP-40, or recovery (F (2,50) = 0.317, *p* = 0.730), or between the sexes (F (1,25) = 0.485, *p* = 0.492) in CRVE. Moreover, no interaction was found between these two factors (F (2,50) = 0.076, *p* = 0.927). Similarly, as for the two previous variables, there were no significant changes between the baseline, LBNP-40, or recovery (F (2,50) = 0.570, *p* = 0.569), or between the sexes (F (1,25) = 0.531, *p* = 0.473) in AVR.

## 4. Discussion

The aim of this study was to investigate changes in retinal microcirculation parameters in healthy male and female participants at rest and during LBNP-induced central hypovolemia. This study showed no significant differences in CRAE and CRVE, either during LBNP or between male and female participants. 

### 4.1. Retinal Microcirculation Measurements

The CRAE showed no significant changes between the baseline, LBNP40, or recovery conditions. Based on these results, the null hypothesis that the LBNP-induced fluid shift does not significantly change the CRAE cannot be rejected, because the measured parameters did not show the expected decrease in arteriolar diameter (decrease in CRAE). Exploratory analyses of the lower-grade negative pressures showed an expected decrease in CRAE from the baseline to LBNP10 and from LBNP10 to LBNP20. However, they also showed a paradoxical increase in CRAE from LBNP20 to LBNP30, reaching a maximum CRAE in LBNP40, then decreasing again in the recovery phase, reaching values close to the baseline measurements. However, none of these changes were significant. Nonetheless, the increased CRAE in LBNP40 (maximal negative pressure during this study) was an unexpected result, because one would expect reflex vasoconstriction and a decrease in CRAE. Because the effect of venule contraction in the hypovolemic state is less well established compared to the arteriolar contraction, these changes are not as unexpected as the changes in CRAE. An expected normal response in a person without cardiovascular problems (such as in our study participants, who were all healthy) would be an increased venous tone, which increases the venous return and thus the preload. This effect appears to be most relevant in the splanchnic venous system, which serves as a pool of blood that can be recruited during hypovolemia [25,26]. It appears that retinal veins may not play a significant role in maintaining a higher venous return during central hypovolemia. 

One possible explanation for the non-significant changes observed in the microcirculatory blood vessel diameter could be attributed to the intricate nature of microcirculatory regulation mechanisms within the central nervous system and the retina. A study conducted by Bill et al. examined the control of the retinal blood flow, which exhibits similar flow control patterns to cerebral perfusion in healthy individuals. Additionally, the retinal microcirculation has the highest density of microvascular pericytes. However, retinal circulation lacks autonomic innervation and depends solely on local vasogenic factors like endothelin, a vasoconstrictor [27]. Similar to the blood–brain barrier, the blood–retina barrier safeguards the retinal cells from alterations in the composition of peripheral blood [27,28]. This suggests that the absence of a correlation between changes in peripheral circulation parameters, like retinal microcirculation, may be attributed to the differential control systems in operation. While the autonomic nervous system primarily regulates the peripheral circulatory system, retinal vessels are predominantly influenced by local vasogenic factors. Therefore, the lack of correlation between the retinal responses during hypovolemia with the observed systemic cardiovascular changes in the present study may be attributed to the absence of autonomic innervation and local vessel control. A study by Koep et al. (2022) further highlights significant differences in the regulation of cerebral and peripheral circulation. The authors suggest that extrapolating the regulation of cerebral vasculature based on peripheral sympathetic nerve activity is not appropriate. Contrarily, cerebral sympathetic nerve activity appears to have an opposing effect compared to peripheral circulation, and its modulation is mediated by changes in intracranial pressure and cerebral blood volume [29]. In the peripheral circulation, sympathetic nerve activity typically results in vasoconstriction of arteries and veins, leading to decreased blood flow. Sympathetic nerve activity in the cerebral circulation can lead to both vasoconstriction and vasodilation, depending on receptor density, distribution, the presence of other vasoactive compounds, and neurotransmitter release. Additionally, the distribution and types of adrenoreceptors in cerebral vessels vary across different regions, indicating region-specific autonomic regulation of cerebral blood flow [29]. These findings reinforce the notion that the regulation of circulation is multifaceted, underscoring the complexity of cerebral blood flow regulation. 

Conversely, reports of data with significant changes in the retinal microcirculation can be found; however, it must be noted that the cardiovascular stress during these studies was more drastic than that of the LBNP protocol used in this study, as most of the obtained data in those studies involved models of septic and hemorrhagic shock. A study conducted by Jurate Simkiene et al. examined changes in retinal microcirculation in patients with sepsis or septic shock compared to healthy individuals. The results showed a significantly higher CRAE during sepsis or in septic shock patients, indicating altered hemodynamic states. However, the CRVE and AVR did not differ significantly between the two groups [19]. This study aligns closely with the methods employed in our LBNP study and suggests that CRAE changes can be observed and correlated with hemodynamic alterations, particularly in septic shock. While there are similarities in the hemodynamic changes observed in septic shock and hypovolemia, it is important to note that they are not identical. In septic shock, the primary mechanism is decreased vascular tone, leading to vasodilation and impaired tissue perfusion. On the other hand, hypovolemia involves a decrease in intravascular volume accompanied by increased vascular tone, which aims to compensate for the reduced blood volume by constricting blood vessels. These distinct mechanisms highlight the different underlying pathophysiologies between septic shock and hypovolemia, despite some overlapping hemodynamic changes. Retinal fluorescein angiography performed by Erikson et al. (2017) in patients with sepsis revealed pathological retinal changes associated with arterial blood flow slowdown, such as vitreous changes, retinal hemorrhages, and fluorescein-leaking microaneurysms. Bilateral involvement was observed in 75% of the cases [20]. These findings further support the notion that retinal microcirculatory changes can be observed in hemodynamic disturbances like sepsis, although fluorescein angiography may provide better visualization compared to measuring vessel diameters. It is important to note that while our study induced a state of hypovolemia rather than a shock-like state, the mentioned studies on septic shock are relevant.

### 4.2. Differences in Males vs. Females

The data analysis carried out in this study did not reveal any significant differences in the retinal vessel diameters (CRAE, CRVE, or AVR) between the male and female participants during the three evaluated time points (baseline, LBNP-40, and recovery). This suggests that sex did not influence the response of retinal vessels to LBNP, contrary to our initial hypothesis. While there was a trend of higher CRAE values in the female participants throughout the study, this difference was not statistically significant. Similarly, the changes in CRAE were identical in males and females during the study protocol. We speculate that the tendency towards a higher arteriolar tone observed in males, indicated by a lower CRAE, may contribute to their enhanced orthostatic tolerance and stronger vasoconstrictor response compared to females. This notion is supported by several studies, including the work of Huxley et al., which suggests that females tend to exhibit an increase in HR rather than SVR in response to orthostatic challenges, whereas males show the opposite pattern [30]. However, it is important to note that our study did not reveal a significant difference in CRAE between males and females. Therefore, our findings do not directly confirm or refute this hypothesis. Further research specifically focusing on the relationship between retinal vessel diameters, arteriolar tone, orthostatic tolerance, and sex differences would be valuable in providing a more comprehensive understanding of these phenomena. While exploratory analyses revealed that the female participants exhibited a tendency towards higher CRVE throughout all the LBNP phases, except for the LBNP10 phase where the male CRVE was higher, these differences did not reach statistical significance. It could be speculated that the presence of more dilated venules in females might lead to a decrease in venous return, resulting in lower preload and decreased orthostatic tolerance, which is typically more common in females. However, since our study did not demonstrate a significant difference in CRVE, we cannot substantiate this hypothesis. Regarding the AVR, it had a tendency to be higher in the female participants, likely attributed to the overall higher CRAE in the females compared to the males. Nevertheless, these differences in AVR were not statistically significant. Although no studies linking retinal AVR to changes in orthostatic tolerance could be found, a lower AVR generally correlates to a higher blood pressure and various cardiovascular risk factors (including BMI, sex, and age). AVR is usually lower in males and in the older population [31,32,33]. 

Further studies are required to gain more insight into potential sex differences in microcirculation during LBNP. Future studies could also include additional data, which could provide valuable insights into the underlying molecular biology and/or mechanisms underlying such changes. For instance, recent bulk transcriptomics studies, which could represent a strong substrate to enforce the role of previously described molecular mechanisms such as the recent studies with PMID: 36290689, PMID: 36490268, and PMID: 32184807, could be incorporated into future measurements and research.

## 5. Limitations

A small sample size could be considered one limitation of our study. However, we do not believe that this is the case, as we carried out a sample size calculation prior to starting the study. Due to difficulties in retinal image acquisition and analysis, complete data sets (baseline, LBNP40, and recovery) from twenty-seven out of the forty-two participants were available for final evaluation. This is in accordance with our sample size calculations, which estimated a minimum participant number of 24 to obtain 80% power. 

We observed a lack of correlation in the retinal responses during hyovolemia with the observed systemic cardiovacular changes. It is possible that the lack of autonomic innervation and local control of retinal vessels could have contributed to these results. Future studies should explore this in more detail. 

In addition, a maximum LBNP of −40 mmHg was used in the present study, which corresponds to the stress experienced while standing upright [3]. Several studies have used LBNP levels of ≥−50 mmHg [4,34]. Greater negative pressures could lead to more drastic changes in retinal microcirculation parameters, which could be more similar to those seen in studies on septic and hemorrhagic shock. 

Finally, the analysis of retinal images was a highly subjective process which frequently required manual adjustments, which is a potential source of error that might have influenced the results. We do not believe this is the case, as the complete data analysis was performed by the same professionally trained grader (AS).

## 6. Conclusions and Future Directions

The present study examined how the LBNP protocol, utilizing a maximum negative pressure of −40 mmHg, influences retinal microcirculation parameters in healthy young individuals. Graded LBNP application did not lead to changes in the retinal microvasculature across the sexes. The present study is the first in the given area that attempted to capture changes in retinal microcirculation caused by central hypovolemia during LBNP. However, further research is needed with higher LBNP levels, including those that can induce pre-fainting (presyncope), to fully understand how the retinal microcirculation adapts during complete cardiovascular collapse (e.g., during hypovolemic shock) and/or during severe hemorrhage. Additionally, future research should include retinal blood flow and velocity measures alongside vessel diameters for a more comprehensive understanding. Finally, integrating additional data in future studies can provide valuable insights into the underlying molecular biology and mechanisms driving these changes.

## Figures and Tables

**Table 1:** Baseline characteristics of the study population. Data are shown as mean ± standard deviation.

Characteristics	Females	Males
N	16	11
Age [y]	22.4 ± 2.7	24.2 ± 4.2
Weight [kg]	58.7 ± 7.3	75.1 ± 8.3
Height [cm]	167.3 ± 6.6	181.6 ± 6.9
BMI [kg/m^2^]	21.0 ± 1.9	22.8 ± 2.4

**Table 2:** The values of the retinal microvascular parameters from three analyzed measurement time points are categorized by sex. The data is presented as the mean ± standard deviation for the central retinal arteriolar equivalent (CRAE), the central retinal venular equivalent (CRVE), and the artery-to-vein ratio (AVR).

Time Point	Baseline		LBNP-40		Recovery	
Sex	Males	Females	Males	Females	Males	Females
CRAE [μm]	140.81 ± 13.26	146.90 ± 11.70	142.18 ± 10.42	149.74 ± 13.84	141.34 ± 13.54	147.41 ± 13.05
CRVE [μm]	203.97 ± 20.52	210.13 ± 23.03	205.44 ± 19.03	210.45 ± 19.39	207.77 ± 18.66	211.40 ± 20.68
AVR	0.69 ± 0.07	0.70 ± 0.07	0.71 ± 0.06	0.71 ± 0.05	0.68 ± 0.07	0.70 ± 0.06

## Data Availability

The datasets generated from the current study are available from the corresponding author upon request.
